# An Efficient Interactive Model for On-Demand Sensing-As-A-Servicesof Sensor-Cloud

**DOI:** 10.3390/s16070992

**Published:** 2016-06-28

**Authors:** Thanh Dinh, Younghan Kim

**Affiliations:** School of Electronic Engineering, Soongsil University, Room 1104, Huyngam Engineering Building 424, Sangdo-dong, Dongjak-Gu, Seoul 06978, Korea; younghak@dcn.ssu.ac.kr

**Keywords:** efficient interactive model, sensor-cloud, IoT cloud, sensing-as-a-service, multiple applications

## Abstract

This paper proposes an efficient interactive model for the sensor-cloud to enable the sensor-cloud to efficiently provide on-demand sensing services for multiple applications with different requirements at the same time. The interactive model is designed for both the cloud and sensor nodes to optimize the resource consumption of physical sensors, as well as the bandwidth consumption of sensing traffic. In the model, the sensor-cloud plays a key role in aggregating application requests to minimize the workloads required for constrained physical nodes while guaranteeing that the requirements of all applications are satisfied. Physical sensor nodes perform their sensing under the guidance of the sensor-cloud. Based on the interactions with the sensor-cloud, physical sensor nodes adapt their scheduling accordingly to minimize their energy consumption. Comprehensive experimental results show that our proposed system achieves a significant improvement in terms of the energy consumption of physical sensors, the bandwidth consumption from the sink node to the sensor-cloud, the packet delivery latency, reliability and scalability, compared to current approaches. Based on the obtained results, we discuss the economical benefits and how the proposed system enables a win-win model in the sensor-cloud.

## 1. Introduction

Over the last few years, studies have exposed the limitations of traditional wireless sensor networks (WSNs) [1,2,3], including sensor system management and the sensing data usage model. Recently, the sensor-cloud architecture has been proposed and has been receiving great interest [1,4,5,6] among researchers in the field of WSNs, as well as cloud computing.

The integration between WSNs and the cloud is motivated by taking advantage of the powerful processing and storage abilities of cloud computing for sensing data. By enabling such an integration, sensor-cloud sensing-as-a-service (SSaaS) can provide sensing data to multiple applications at the same time, instead of the current sensing data usage model for a dedicated application. The sensor-cloud can help improve the utilization of sensor resources, as well as sensor management and functions as an interface between physical sensor networks and the cyber world [1,7]. For those reasons, the sensor-cloud is being viewed as a potential substitute of traditional WSNs [1,7].

In the sensor-cloud architecture [1,4,5], the application model is changed as follows:

- Physical sensors perform sensing and forward sensing data to the sensor-cloud.
- The sensor-cloud virtualizes sensor nodes as virtual sensors and provides sensing-as-a-service to users and applications.
- Applications/users buy sensing services on demand from the sensor-cloud.

One of main targets of the sensor-cloud is to enable a single physical sensor network to provide sensing services to multiple applications at the same time and enable users/applications to request sensing services on demand based on their needs (i.e., by allowing applications to specify their region of interest and sensing frequency [1,4,5]). Although a number of studies have been initially investigated, there is still a lack of a specific and efficient scheme on processing the down-stream traffic of application requests from cloud-to-sensors (C2S), in other words on how the sensor-cloud should process application requests and interact with physical sensors to efficiently support multiple applications on demand, toward the above targets.

Without optimizing application requests as in the current sensor-cloud, only applications having the same requirements (i.e., sensing interval, latency) can reuse the sensing data of each other. Therefore, any dedicated application request with a new requirement will be sent to physical nodes. To support multiple applications with different requirements, a physical sensor may have to run multiple schedules (i.e., multiple dedicated sensing intervals) for different applications. In several existing studies, dedicated sensing requests [8,9] are used. This approach is obviously inefficient for constrained resource devices like sensors, as they have to process a great number of requests from different applications with possibly high data redundancy. Some other sensor-cloud designs provide only limited sensing services within a fixed sensing rate [7,10], which may not satisfy all applications.

Allowing on-demand sensing services on the sensor-cloud poses enormous technical challenges. For example, on-demand sensing services may lead to dynamic operations at physical sensor nodes. In particular, when a new sensing request with a new sensing requirement is sent to physical WSNs, physical nodes have to add or update their sensing tasks to meet the requirements of all applications. On-demand sensing requests from applications may overuse physical sensor networks if request optimization is not considered properly, thus incurring high operational cost for sensors’ owners. In addition, how physical sensors can perform sensing tasks efficiently to satisfy all applications at the same time is also an open question. For those reasons, an efficient interactive model requires the involvement of both the sensor side and the cloud side.

To address the above challenges, we identify the following requirements for the integration between WSNs and the sensor-cloud: (1) application requests’ optimization at the sensor-cloud is required to minimize the number of requests sent to physical WSNs; (2) physical sensors should have the ability to cope with dynamic conditions and should interact with the sensor-cloud to automatically adapt (i.e., its scheduling, etc.) to network condition changes to optimize their operations.

In this paper, we first propose an efficient interactive model for applications, the sensor-cloud and physical sensor networks to support on-demand periodic sensing services for multiple applications. In the model, the sensor-cloud processes applications’ requests and performs request aggregation to minimize the number of requests sent to physical sensors. Upon receiving requests, physical sensors update their sensing service to meet the requirements of all applications and automatically adapt their operations upon the changes to minimize energy consumption.

For illustration, we describe the interactive model using sensing requests with different sensing interval requirements as an example, but the model can be generalized for any application requirement, such as packet latency, reliability, etc. In particular, for sensing requests with different sensing interval requirements, the sensor-cloud aggregates all applications’ requests and selects an optimal consolidated sensing interval for a set of sensors, which minimizes the number of sensing and the number of data packet transmissions of physical sensor nodes while satisfying all applications’ sensing interval requirements.

Whenever a new consolidated sensing interval is found by the aggregator on the sensor-cloud, the aggregator sends a sensing update request to a physical sensor manager, which will forward the request to corresponding physical sensors. The physical sensors receive the request and update their sensing interval accordingly to meet the requirements of all applications. Because the sensing update request implicitly notifies about changes in the network traffic condition, the physical sensor nodes automatically adapt their scheduling parameters to optimize energy consumption. Requests from new applications, which the aggregator determines that the current consolidated sensing interval still satisfies, are hidden from physical sensors to save energy. As a result, data reusability is transparent to the sensor-cloud users, and sensing redundancy is reduced.

We conduct extensive analysis and experiments to evaluate the performance of the proposed model in terms of the energy consumption of physical sensors, the bandwidth consumption from the sink node to the sensor-cloud, the packet delivery latency, reliability and scalability. We compare the performance of the proposed system with (1) a dedicated application request model with a traditional in-network aggregation mechanism [11] and (2) a dedicated application request model with a multi-task optimization scheme [12] . Results show that the proposed on-cloud request aggregation is significantly more efficient than the traditional in-network aggregation and multi-task optimization, so it can be a promising approach to complement the in-network aggregation to save network resources. The proposed interactive model helps reduce the cost for both sensor owners (i.e., energy consumption of physical sensors) and sensor-cloud providers (i.e., bandwidth consumption). In addition, by achieving a high scalability, the model enables a single physical sensor network to efficiently provide sensing services to a great number of applications with low cost, which benefits cloud providers and sensor owners. As a result, the model potentially reduces the price of sensing services per application, which benefits application owners and users, thus enabling a win-win model in the sensor-cloud.

In summary, this paper makes the following contributions.

- We propose an efficient interactive model for the sensor-cloud, which enables the sensor-cloud to provide on-demand sensing services to multiple applications at the same time.
- We design an efficient request aggregation scheme on the sensor-cloud to minimize the number of requests sent to physical sensor nodes and an efficient request-based adaptive low power listening protocol for physical sensor nodes to optimize sensors’ energy consumption.
- Through our comprehensive experimental studies, we show that the proposed system achieves a significant improvement in terms of the energy consumption of sensor nodes, the bandwidth consumption of sensing traffic, the packet delivery latency, reliability and scalability, compared to the state-of-the-art approaches.

The rest of this paper is organized as follows. Section 1 discusses related works. Section 2 presents the proposed interactive model. Section 3 describes the request-based adaptive protocol. Section 4 gives details about the system implementation and shows the evaluation results. Finally, in Section 5, we discuss the limitations of the current work, possible extensions in this topic for future works and conclude the paper.

## 2. Related Work

Recently, the sensor-cloud has been proposed as a promising architecture, which has been receiving great interest [1,5,6] among researchers. Although there are several main architecture designs of the sensor-cloud that have been proposed [1,5,6], their basic design is quite similar. In particular, physical sensors perform sensing and forward sensing data to the sensor-cloud. The sensor-cloud provides sensing services to multiple users/applications [13] through virtual sensors. The sensor-cloud virtualizes physical sensor nodes into virtual sensors. A virtual sensor is an emulation of a physical sensor on the sensor-cloud. The sensor-cloud uses virtual sensors to provide sensing services to users/applications with a customized view. Users/applications buy sensing services on demand from the sensor-cloud. Virtual sensors obtain data from underlying physical sensors and contain metadata about the corresponding physical sensors, as well as applications currently holding those virtual sensors.

A number of initial research works have been conducting toward a more detailed design for the sensor-cloud using different approaches. We categorize related works based on their approach.

The sensor-cloud obviously provides many opportunities to develop sensing services [14,15,16,17]. In [14], Dinh and Younghan Kim propose to exploit the sensor-cloud for smart cities. In particular, a location-based sensor-cloud model is designed to support government officers on managing parking violation efficiently. In [15], Giovanni et al. also propose a framework, namely Stack4Things, for smart city applications. However, in Stack4Things, a device-oriented approach is used with fog computing, instead of the location-centric approach as used in [14]. Giancarlo et al. [16,17] propose to integrate the cloud computing platform with a body sensor network (BSN), where a multi-tier application-level architecture is used to allow a rapid development of BSN applications. In fact, there are still many challenges [1,5,18] that need to be investigated for an efficient integration between WSNs and the cloud. For different application fields, such as healthcare [19], some unique requirements and challenges may exist. Enabling efficient on-demand sensing-as-a-service is one of the main challenges that need to be taken into account for WSNs and cloud integration.

Data processing is an important aspect of the sensor-cloud. In [20,21], the authors investigate on upstream data processing optimization [22,23] in the sensor-cloud using different techniques, including compression, filtering, encryption, decryption, etc. Jun et al. [24] propose a queuing model and an efficient congestion control, namely random early detection-based (RED-based), mechanism for upstream sensing data to improve data transmission from sensors to the cloud. In [25], Samer et al. propose a data prediction model to improve data transmission and data processing in the sensor-cloud. In particular, the model is built within sensors and run by the sensor-cloud to generate data so that a large amount of data transmission at the sensor nodes is reduced to save energy. Barbaran et al. [26] use virtual channels to simplify the integration of WSNs in the cloud. Virtual channels are used to exchange messages between every single device and the cloud to achieve highly reconfigurable and self-managed features.

In the sensor-cloud, traditional data collection schemes can also be extended. In particular, a sensor node may have multiple options for gateways to forward its data toward the cloud instead of a dedicated sink as in traditional WSNs. Chatterjee et al. [7] and Misra [9] improve the integration between the sensor-cloud and physical nodes by proposing schemes to select optimal gateways for sensors to forward sensing data to the cloud. The studies demonstrate that selecting good gateways can significantly reduce sensing data forwarding overhead toward the sensor-cloud.

As sensors are normally deployed with a high density, how the sensor-cloud selects a number of physical sensor nodes based on virtual sensors to execute a task is an interesting topic. The QoS-aware sensor allocation mechanism [27] enables the sensor-cloud to allocate an optimal set of sensors for a particular task with the awareness of quality of service. Having a similar target, however, Sen et al. [28] implement a collaborative platform for sensor allocation based on the sensors’ coverage. Zhu et al. [29] believe that some nodes may perform a task better than others and propose to use trust for sensors and data center selection. In particular, sensors and data centers with a high trust value are selected to guarantee quality of services.

In [30,31,32], various caching techniques are proposed to conserve the network resources of sensor nodes when they are integrated with the sensor-cloud. The caching mechanisms are normally supported by the cloud and deployed at gateways. In the studies, the caching mechanisms are designed in a flexible way for various rates of changes of the physical environments. By interacting with sensor nodes, the cloud can be used to estimate parameters for sensor nodes [33] to improve their performance.

Under an assumption that a number of distributed data centers can be built to provide sensing services, Chatterjee et al. [34] optimize sensing service transmission and sensing management by de-compositing sensing data to the closest cloud data center and scheduling a particular data center to congregate data from virtual sensors. In [8,35], the authors use a reversed approach where the cloud is used as a controller to control the sleep schedule of sensors based on the location of users.

A sensor-cloud is actually a cloud of virtual sensors, which are mapped with physical nodes, so an efficient management scheme for virtual sensors is required. Ojha et al. [35,36] propose an efficient virtualization scheme for physical sensor nodes and seek for an optimal location-based composition of virtual sensors. The scheme consists of two parts (1) for sensors within the same geographic region (CoV-I) and (2) spanning across multiple regions (CoV-II).

In [37,38,39,40,41,42,43], different pricing models and usage models for the sensor-cloud are proposed. All pricing models have a similar approach, which is that the price of a sensing service is normally proportional to the sensing quality (i.e., sensing frequency or interval between two consecutive sensings). The studies show that the sensor-cloud approach has the potential for various types of applications from healthcare to smart cities.

Although one of the main targets of the sensor-cloud is to enable SSaaS on demand, there is still a lack of a specific and efficient interactive model toward the above targets. This paper investigates an efficient interactive model between the sensor-cloud and sensor nodes to fill the gap.

## 3. The Proposed Interactive Model

In this section, we present our proposed interactive model, which efficiently supports on-demand sensing services to multiple applications at the same time. The model enables the sensor-cloud to provide on-demand sensing services where applications can send sensing requests with their own choice of sensing parameters based on the applications’ needs and prices. The sensor-cloud processes applications’ requests and performs request aggregation to minimize the number of requests sent to physical sensors while ensuring that their sensing services meet the requirements of all applications. To make the model easy to understand, we present the model using sensing requests with different sensing interval requirements as an example. However, the model can be generalized for any application requirement, such as packet latency, reliability, etc. A list of symbols used in the model is given in Table 1.

### 3.1. Sensor-Cloud Modeling

**Figure 1:**
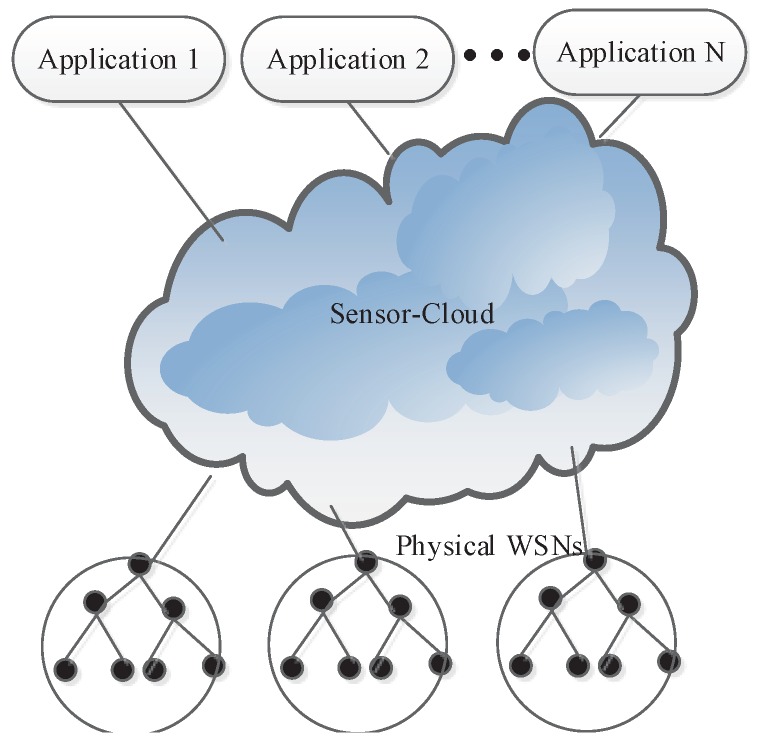
Location-based IoT-cloud integration.

We first present the sensor-cloud model for physical WSNs and cloud integration. The basic architecture of the sensor-cloud [1,4,5] is illustrated in Figure 1 and is modeled as follows.

**Physical Wireless Sensor Networks.** A wireless sensor network consists of physical sensor nodes. Each sensor node is characterized by the following properties: ID, type $i_{\tau }$, state *ς*, sensing interval $I_{se}$, ownership *O* and a set of scheduling parameters *S*.

**Definition 1.**  *Each physical sensor node i is associated with a sensor type* $i_{\tau }$*, with* $i_{\tau }\in \tau =\left\{\tau_{1},\tau_{2},...,\tau_{N}\right\}$*, where τ is a set of N registered sensor types of the sensor-cloud.*

**Definition 2.**  *During the lifetime, a sensor node may be in the active state (denoted by one) or inactive (denoted by zero). The state of a node i is denoted by* $i_{\varsigma }$.

**Definition 3.**  *Each sensor node belongs to an owner θ who contributes sensing services to the sensor-cloud. Note that there may be a set of multiple WSN owners* Θ *within a sensor-cloud.*

**Definition 4.**  *Each sensor node i performs sensing in every interval of* $i_{I_{se}}$ *and transmits sensing data to the cloud. Note that in conventional approaches, a sensor may perform sensing at a fixed rate or at multiple rates for different applications. In our interactive model, the sensors’ sensing rate is determined by the cloud and dynamically changed upon requests from the cloud.*

**Definition 5.**  A physical sensor node operates with a set of scheduling parameters S, which determine how long a node should sleep and how long it should remain awake every cycle. In our interactive model, based on the interactions with the sensor-cloud, physical sensor nodes optimize S to minimize energy consumption while satisfying all applications.

By our definition, a physical sensor *i* is modeled as follows.

$i=\left(i_{ID},i_{\tau },i_{\varsigma },i_{\theta },i_{I_{se}},i_{S}\right),i_{\tau }\in \tau ,i_{\theta }\in \Theta$.

**Cloud C.** The sensor-cloud virtualizes physical sensors, maps them into virtual sensors and provides sensing-as-a-service to users/applications [1]. In other words, the sensor-cloud is composed of virtual sensors built on top of physical sensors. A cloud *c* is characterized by the following properties: ID, resources, QoS and price options. The cloud *c* may provide sensing services for a set of *τ* sensor types from Θ WSN owners. Based on a pricing model for the sensor-cloud [4], the price of a sensing service is normally proportional to the sensing quality (i.e., sensing frequency or the interval between two consecutive sensing). For example, a user has to pay a higher price if he or she requests a higher sensing frequency (i.e., shorter sensing interval request). The reason is that for a higher sensing frequency, more resources are required in the physical sensor networks and cloud infrastructure. Note that this paper does not consider a selective model for clouds, so we do not present the properties of a cloud in detail.

**Definition 6.**  A virtual sensor is an emulation of a physical sensor and provides a customized view to users for sensing data distribution transparently [1]. In fact, virtual sensors are implemented as software images of the corresponding physical sensors on the cloud. Virtual sensors contain metadata about the corresponding physical sensors for mapping purposes and applications holding the virtual sensors [1].

**Application.** An application *α* is characterized by the following properties: ID, a set of sensor data types of interest, region of interest and QoS requirements (i.e., sensing interval).

**Definition 7.**  *An application α may be interested in a set of sensor data types* $\alpha_{SI}$ *for its operations. As the target of the sensor-cloud is to enable applications to be transparent regarding the types of sensors used [1,4,5], we define only a set of sensor data types of interest for applications. We later provide a function to map* $\alpha_{SI}$ *of an application α to a set of sensor types τ.*

**Definition 8.**  *An application α is normally deployed to work in a limited region, called a region of interest* $\alpha_{RI}=\left(L_{1},L_{2},L_{3},L_{4}\right)$*. The region of interest consists of the location of four points that bound the region. The sensor-cloud should manage the locations of physical sensor nodes and map them to virtual sensors on the cloud and the regions of interest of applications [1].*

**Definition 9.**  *Each application α may request different QoS requirements* $\alpha_{QoS}$ *for sensing data, such as delay or sensing frequency, namely dedicated sensing requests. The sensor-cloud is designed to provide sensing services to multiple applications, instead of a dedicated application, as in the traditional WSNs. In this work, we use sensing frequency (i.e., sensing interval) requirements to illustrate the model. A sensing interval requested by a particular application is called a dedicated sensing interval.*

An application *α* is modeled as follows.

$\alpha =(\alpha_{ID},\alpha_{SI},\alpha_{RI},\alpha_{QoS})$

**Definition 10.**  *A dedicated sensing interval* $I_{se}$ *is the sensing frequency requested by a specific application.*

How the sensor-cloud can efficiently handle dedicated sensing interval requests from different applications and how physical sensors can schedule their operations efficiently while satisfying all applications’ requests on demand are critical questions that need to be solved. In the next section, we describe an efficient interactive model in detail to address those questions.

### 3.2. An Efficient Interactive Model for the Sensor-Cloud (C2S)

**Figure 2:**
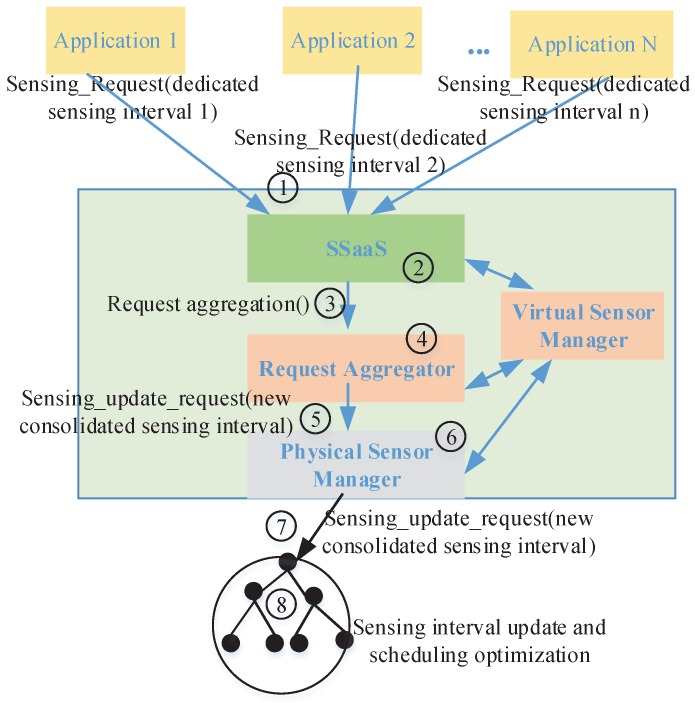
The proposed interactive model for the sensor-cloud.

Figure 2 shows our proposed interactive model for the sensor-cloud. In this version, the model focuses on the down-stream traffic of application requests from cloud-to-sensors (C2S). The up-stream traffic of sensing data packets from sensors-to-cloud (S2C) will be investigated in our future work.

In the C2S model, the sensor-cloud plays the role as a the middleware between applications and physical sensors. In WSNs’ deployment phase, deployed physical sensors are registered with the sensor-cloud. The physical sensors are then mapped into virtual sensors on the sensor-cloud [35]. Virtual sensors are managed by the virtual sensor manager (VSM), as shown in Figure 2.

We model a function that maps a physical sensor or a set of physical sensors *ζ* to a virtual sensor or a set of virtual sensors *γ* as follows.

(1) $$f_{phy->vir}\left(\zeta \right)=\gamma$$

Note that a mapping mechanism [36] is out of the scope of this paper.

Sensing data collected from physical sensors are stored at virtual sensors and distributed by the sensor-cloud. The sensor-cloud then provides sensing-as-a-service (SSaaS) to different applications based on virtual sensors [1,4,5].

This paper investigates the processes of the sensor-cloud when it receives application requests and how it interacts with physical nodes. A virtual sensor and its corresponding physical sensor may serve multiple applications at the same time. Therefore, we propose an efficient interactive model to minimize the number of application requests sent to physical nodes while the requirements of all applications are satisfied.

The interactive model is described as follows.

1. A buyer (i.e., application owner) buys a sensing service of the sensor-cloud for a new application *α*. The application sends a request to the SSaaS of the sensor-cloud for a sensing service. Based on the application’s demand and budget [4], the application specifies the following parameters: (1) a set of sensor data types of interest $\alpha_{SI}$; (2) a region of interest $\alpha_{RI}$; and (3) requirements (i.e., sensing interval (i.e., $I_{se}$)) $I_{se}^{\alpha }$. A request including those parameters is sent to the SSaaS.

2. First, the SSaaS needs to map the sensor data types of interest (SI) of the application $\alpha_{SI}$ to a set of actual sensor types (ST) $\tau_{\alpha }^{*}\subset \tau$. The mapping function is modeled as follows.

(2) $$f_{SI->ST}\left(\alpha_{SI}\right)=\tau_{\alpha }^{*}=\left(\tau_{j}:\tau_{j}\in \tau \right)$$

Based on the application’s actual sensor types of interest and region of interest, the sensor-cloud requests the VSM to allocate a set of virtual sensors $\gamma_{\alpha }^{*}$ to provide sensing services for the application. The allocation function is modeled as follows.

(3) $$f_{viralloc}\left(\alpha_{RI},\tau_{\alpha }^{*}\right)=\gamma_{\alpha }^{*}=\left(\gamma_{j}:\gamma_{j->type}\in \tau_{\alpha }^{*}\right)\;and\;\gamma_{j->location}\in \alpha_{RI}$$

3. The request is then forwarded to the request aggregator for optimization.

4. The aggregator processes the request and determines whether or not updates in corresponding physical sensors are required (i.e., changing the sensing rate at physical nodes) to satisfy the requirements of all applications, including the new one. The aggregator determines that an update is required or not based on the requests of applications and the current configuration information of the virtual sensors managed by the VSM. If an update is required, the aggregator determines a new consolidated sensing interval $I_{se}^{c-new}$ for the corresponding physical nodes.

5. If an update is required, the aggregator sends a sensing update request containing the new consolidated sensing interval $I_{se}^{c-new}$ (i.e., $sensing_{u}pdate_{r}equest\left(I_{se}^{c-new}\right)$) to the physical sensor manager (PSM).

6. The PSM reversely maps the set of virtual sensors $\gamma_{\alpha }^{*}$ to a set of corresponding physical sensors *ζ*. We model the reserved mapping function as follows.

(4) $$f_{vir->phy}\left(\gamma \right)=\zeta =f_{phy->vir}^{-1}\left(\zeta \right)$$

7. The PSM then forwards the sensing update request to the corresponding physical sensor nodes. The detailed design of the protocol for PSM sending requests to physical sensors will be studied in future work.

8. Upon receiving the sensing update request, the physical nodes update their sensing interval to meet the requirements of the new application and all existing applications. The physical nodes then optimize their scheduling parameters to minimize energy consumption.

An aggregation mechanism for the aggregator to aggregate application requests is proposed in the next section.

### 3.3. Application Request Aggregation Scheme

Without optimizing application requests as in the current sensor-cloud, only applications with the same requirements (i.e., same sensing interval) can share sensing data. As a result, any dedicated application request with a different requirement will be forwarded to sensor nodes. To provide SSaaS to multiple applications with different requirements simultaneously, a sensor node may have to operate with multiple schedules. This results in a high resource consumption at the physical sensor and a high bandwidth consumption, as well as storage usage at the sensor-cloud, while the sensing data may be highly redundant. As constrained resource sensor nodes are required to run multiple tasks at the same time, the scalability of the system is limited. For that reason, the sensing service cost per an application may be not competitive.

In practice, applications with different sensing interval options can still share the same sensing dataset in many cases as long as the sensing frequency (i.e., sensing interval) of the dataset satisfies all applications’ requirements. For example: (1) applications with a packet delivery reliability requirement of 90% normally accept sensing data with a higher reliability of 98%; (2) applications normally accept more frequent sensing (i.e., sensing interval of 5 s) than their requests (i.e., sensing interval of 10 s); more frequent sensing is only avoided for efficiency reasons [44]. For that reason, there may exist a single consolidated sensing parameter for sensor nodes to satisfy all applications’ requirements, thus enabling constrained sensor nodes to run a single sensing task while serving multiple applications at the same time.

The request aggregator proposed in the interactive model has a role to determine whether a sensing parameter satisfies a set of applications or not. If a single sensing parameter satisfies a set of applications, the sensor-cloud can use the same sensing dataset with that sensing parameter to distribute to all applications appropriately (i.e., all applications may receive the same set of sensing data or some applications may only need to receive a part of the data set, depending on the needs of the applications and the distribution policies of the sensor-cloud).

#### 3.3.1. The Request Aggregator

When the aggregator receives a new request from a new application for the SSaaS for a set of virtual sensors, it first queries the information of the virtual sensors from the VSM and classifies the virtual sensors used by a set of applications, including the new application.

The virtual sensor manager manages all information of the virtual sensors, including metadata, applications that are using the sensing services provided by the virtual sensors and their current consolidated sensing interval $I_{se}^{c}$.

We assume a set of virtual sensors *γ* that are used by a set of applications *A*, including the new application. Their current consolidated sensing interval is $I_{se}^{c}$. Each application α∈A requests a dedicated sensing interval of $I_{se}^{\alpha }$. The new application $\alpha_{new}$ requests a dedicated sensing interval $I_{se}^{\alpha -new}$. The aggregator now aggregates requests from all applications in the set *A* to determine an optimal sensing interval for the sensors, which satisfies the requirements of all applications. The application request aggregation procedure at the aggregator is presented in Algorithm 1.

**Algorithm 1** Application request aggregation procedure.**INPUT:**
$\gamma ,A,I_{se}^{c},\alpha_{new}$**OUTPUT:** updating-flag, new $I_{se}^{c}$ if updating-flag = 1**Initialize:** updating-flag = 0, x←∞**Repeat** x = aggregation($A,\alpha_{new})$ **if**
$isNew(x,I_{se}^{c})$
**then**  $I_{se}^{c}=x$;  updating-flag = 1  **return** updating-flag; **end**
**if** **return** updating-flag; **UNTIL** there is no new application request

The aggregator aggregates sensing interval requests of all applications together and determines a consolidated sensing interval. If the returned updating-flag is one, this means that the aggregator finds a new consolidated sensing interval for all applications, including the new application. As a result, the aggregator creates a sensing update request with the new consolidated sensing interval and sends the request to the PSM, which is then sent to corresponding physical sensor nodes. If the return updating-flag is zero, this means that the current consolidated sensing interval satisfies all applications, including the new one. As a result, the new application request is hidden from the physical sensors, and no sensing update is required for physical sensors to serve the new application.

#### 3.3.2. The Aggregation Function

The design of the aggregation function, as used in the procedure above, depends on the objectives of the sensor-cloud and the request parameters (i.e., sensing interval, latency, reliability) of applications. We here define an aggregation function $f_{agg}$ for a sensing interval parameter as follows. The objective is to find a consolidated sensing interval for a set of physical sensor nodes that minimizes the number of sensing samplings and the number of packet transmissions of the physical nodes while the sensing interval requirements of all applications are still satisfied. According to the observation in [44] as mentioned above, we have the following definition.

**Definition 11.**  *A sensor node satisfies a sensing request of an application α if the sensor performs sensing and sensing data transmission at least every* $I_{se}^{\alpha }$ *seconds, where* $I_{se}^{\alpha }$ *is the dedicated sensing interval requested by α.*

From Definition 11, we have the lemma as follows.

**Lemma 1.**  *A sensor node with its actual sensing interval* $I_{se}$ *satisfies an application α with a requested sensing interval of* $I_{se}^{\alpha }$ *if and only if* $I_{se}\leq I_{se}^{\alpha }$.

**Proof.**  For $I_{se}\leq I_{se}^{\alpha }$, it is clear that the node satisfies the sensing request of *α*, proved using Definition 11.

If $I_{se}>I_{se}^{\alpha }$, the node performs sensing every $I_{se}$ second, which is longer than the application requirement of $I_{se}^{\alpha }$. According to Definition 11, the node does not satisfy the requirement of *α*.

Given a set of N dedicated sensing intervals $I_{se}^{A}=\left(I_{se}^{\alpha_{1}},I_{se}^{\alpha_{2}},...,I_{se}^{\alpha_{N}}\right)$ requested by a set of N applications $A=(\alpha_{1},\alpha_{2},...,\alpha_{N})$ for a set of virtual sensors $\gamma^{*}$, the purpose of the aggregation function is to find a consolidated sensing interval $I_{se}^{c}$ as follows.

*aggregation(*$I_{se}^{A}$) –> $I_{se}^{c}$

so that:

$I_{se}^{c}$ *satisfies* $\alpha_{i}\forall \alpha_{i}\in A$.

We denote $I_{se}^{min}=\min\left(I_{se}^{\alpha_{1}},I_{se}^{\alpha_{2}},...,I_{se}^{\alpha_{N}}\right)$ as the minimum sensing interval among dedicated sensing intervals of the applications in A, and $I_{se}^{min}$ is the dedicated sensing interval of an application $\alpha_{m}\in A$.

**Lemma 2.**  *A consolidated sensing interval* $I_{se}^{c}$ *of* $\gamma^{*}$ *satisfies all applications in A if and only if* $I_{se}^{c}\leq I_{se}^{min}$.

**Proof.**  The consolidated sensing interval $I_{se}^{c}$ is the actual sensing interval of the sensor nodes $\in \;\gamma^{*}$.

For $I_{se}^{c}\leq I_{se}^{min}$, $\forall \alpha_{i}\in A,I_{se}^{c}\leq I_{se}^{\alpha_{i}}$. According to Lemma 1, the sensor nodes perform sensing with an interval of $I_{se}^{c}$ satisfying the application $\alpha_{i},\forall \alpha_{i}\in A$.

For $I_{se}^{c}>I_{se}^{min}$, $\exists \alpha_{m}\in A:I_{se}^{c}>I_{se}^{\alpha_{m}}$. According to Lemma 1, the sensor nodes performing sensing with an interval of $I_{se}^{c}$ do not satisfy at least one application $\alpha_{m}\in A$. As a result, $I_{se}^{c}$ does not satisfy all applications in A.

The final objective of the aggregation function is to find a consolidated sensing interval $I_{se}^{c}$ that helps minimize the number of sensing $N_{s}$ and the number of data transmissions $N_{packet}$ for physical nodes corresponding to $\gamma^{*}$ in a time period *T*, while satisfying all applications in A. For simplification, we assume that after performing a sensing task, a sensor node creates and transmits a sensing data packet toward the sensor-cloud. Because *T* is a constant, we simplify the above problem by finding the maximum value of $I_{se}^{c}$.

**Definition 12.**  *In a time period T, the number of sensings* $N_{s}$ *of a sensor node is* $N_{s}=T/I_{se}$*, where* $I_{se}$ *is the actual sensing interval of the node. We then have the number of data packet transmissions of the node generated by itself to be* $N_{packet}=N_{s}$.

Based on Lemma 1 and Lemma 2, the solution is found using the following theorem.

**Theorem 1.**  *The consolidated sensing interval* $I_{se}^{c}$ *that minimizes the number of sensing* $N_{s}$ *and the number of data transmissions* $N_{packet}$ *in any time period T for physical nodes corresponding to* $\gamma^{*}$ *while satisfying all applications in A is equal to* $I_{se}^{min}$.

**Proof.**  If every node in $\gamma^{*}$ operates with the same $I_{se}^{c}=I_{se}^{min}$, the number of sensing and data packet transmissions of each node is:

(5) $$N_{s}^{c}=N_{packet}^{c}=T/I_{se}^{c}=T/I_{se}^{min}$$

If a node maintains multiple sensing schedules for different applications in A based on their dedicated sensing intervals [12,41], we calculate the number of sensings $N_{s}^{d}$ and the number of data packet transmissions $N_{packet}^{d}$ for each node as follows. We denote $N_{s}^{\alpha_{i}}$ and $N_{packet}^{\alpha_{i}}$ as the number of sensings and the number of data packet transmissions of the node for an application $\alpha_{i}\in A$, respectively.

(6) $$N_{s}^{d}=N_{packet}^{d}=\sum_{i=1}^{N}\left(N_{s}^{\alpha_{i}}\right)=\sum_{i=1}^{N}\left(T/I_{se}^{\alpha_{i}}\right)=N_{s}^{\alpha_{m}}+\sum_{i}^{A\backslash \alpha_{m}}\left(N_{s}^{\alpha_{i}}\right)$$

For the application $\alpha_{m}$ that has a sensing interval equal to $I_{se}^{min}$, we have:

(7) $$N_{s}^{\alpha_{m}}=N_{packet}^{\alpha_{m}}=T/I_{se}^{min}$$

In addition, we have:

(8) $$\sum_{i}^{A\backslash \alpha_{m}}\left(N_{s}^{\alpha_{i}}\right)\geq 0$$

From Equations (7) and (8), we have:

(9) $$N_{s}^{d}=N_{packet}^{d}\geq T/I_{se}^{min}$$

Compare Equations (5) and (9); we conclude that a node that maintains multiple sensing schedules with dedicated sensing intervals of the applications has to perform sensing and data packet transmission more frequently than using a consolidated sensing interval selected by our model.

With the selected consolidated sensing interval following Theorem 1, we have:

(10) $$I_{se}^{c}=I_{se}^{min}\leq I_{se}^{min}$$

According to Lemma 2, sensor nodes running with the selected consolidated sensing interval $I_{se}^{c}$ satisfy all applications in A.

For any sensing interval of $I_{se}^{c^{\prime }}<I_{se}^{min}$ to satisfy all applications in A according to Lemma 2, the number of sensings $N_{s}^{c^{\prime }}$ and the number of data packet transmissions $N_{packet}^{c^{\prime }}$ in a period of time T are calculated as follows.

(11) $$N_{s}^{c^{\prime }}=N_{packet}^{c^{\prime }}=T/I_{se}^{c^{\prime }}>T/I_{se}^{min}=T/I_{se}^{c}$$

From Equations (5)–(11), Theorem 1 is proven.

Based on the proven Theorem 1, we have the aggregation function for sensing interval requests as follows.

(12) $$aggregation\left(I_{se}^{A}\right)=\min\left(I_{se}^{\alpha_{1}},I_{se}^{\alpha_{2}},...,I_{se}^{\alpha_{N}}\right)$$

The aggregator uses the above aggregation function to determine the optimal consolidated sensing interval for a set of sensor nodes, which minimizes their numbers of sensing and sensing packet transmissions while satisfying the sensing requests of all applications.

## 4. A Sensing Update Request-Based Adaptive Low Power Listening Protocol

Another objective of our interactive model is to minimize the energy consumption of sensor nodes while satisfying the sensing requests of all applications. Therefore, in addition to the usage of the optimal consolidated sensing interval determined by the aggregator above, a physical sensor also needs to adapt its wakeup schedule based on the updates of the consolidated sensing interval. We assume physical sensors running with a low power listening protocol (LPL) [45,46,47], which is one of the most popular energy-efficient protocols deployed for WSNs. In this section, we propose a sensing update request-based adaptive low power listening protocol (SLPL) for sensor nodes. A part of the protocol is based on our previous work [46].

Each time a sensing update request is received from the PSM, the physical sensors change their sensing interval accordingly. This means that the traffic produced by each node and incoming traffic to each node are also changed. To optimize energy consumption, the sensors should optimize their low power listening parameters accordingly. For energy efficiency, we propose two adaptive modes for the adaptive protocol: active adaptive mode and lazy adaptive mode. The active mode is used to enable a sensor to adapt its parameters quickly upon changes in network traffic. The lazy mode minimizes the number of traffic samplings to save energy when the network traffic is stable.

### 4.1. Adaptive LPL Triggering Event

When a node receives a sensing interval update request, the traffic network condition at the node is implicitly notified to change. The sensor then switches its adaptive mode to active mode. The sensing interval update request triggers such an event. Note that not only physical nodes that are requested to update their sensing interval, but also intermediate nodes that forward the request also switch to the active mode. The reason is that sensing interval changes at requested nodes also affect incoming traffic at their intermediate nodes.

When a sensor observes that its total traffic becomes stable in a period of *ψ* cycles, it switches its adaptive mode to the lazy mode to save energy.

### 4.2. Active Mode

In the active mode, a node measures the incoming data rate more frequently to quickly observe how much its total traffic changes to adapt its LPL parameters accordingly. We assume that a traffic measurement interval of a sensor in the active mode is $\omega_{active}$, which is shorter than that of the lazy mode.

### 4.3. Lazy Mode

In lazy mode, a node lazily performs data rate measurement to save energy. The reason is that when the network traffic becomes fairly stable and no nodes are requested to update their sensing interval, LPL parameter adaptation is normally insignificant. The benefit of such a parameter adaptation may be not considerable compared to the traffic measurement cost. In addition, the lazy mode is applied to achieve the stability of the system. We assume that the traffic measurement interval of a sensor in lazy mode is $\omega_{lazy}$, which is longer than that of the active mode.

### 4.4. Revisiting LPL Operations

**Figure 3:**
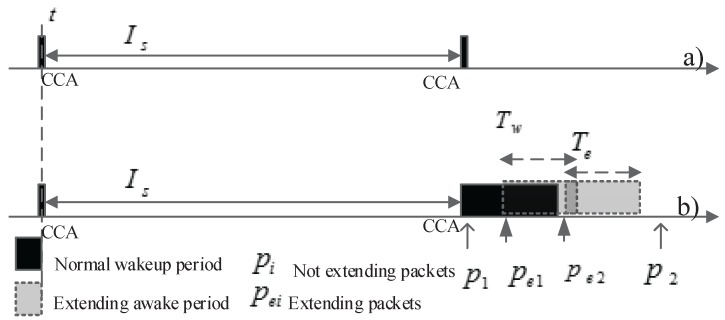
Low power listening protocol (LPL) operations: (**a**) CCA checks and (**b**) CCA checks with received packets.

Low power listening (LPL) [45,46,47] is a common mechanism that has been greatly explored in designing energy-efficient MAC protocols. Although there are several different LPL implementations, their basic design is quite similar. In LPL, a node periodically wakes up (after a sleep interval $I_{s}$) to perform receive checks (CCA), as illustrated in Figure 3a. If there is no channel activity detected, the node then turns off its radio. If the channel is busy, the node wakes up fully and remains active for a wakeup period $T_{w}$ to listen for incoming packets, as shown in Figure 3b. In Figure 3b, the node receives several packets (i.e., $p_{1},p_{e1},p_{e}2$). Before transmitting a packet, senders send preambles until their receiver wakes up. For each packet received, a receiver extends its active time by an extended period $T_{e}$ (Figure 3b), because there may be more than one incoming packet or sender. However, whether a packet is an extending packet or not depends on the packet received time. This will be further analyzed in the next subsections.

### 4.5. Motivations for the Sensing Update Request-Based Adaptive LPL Protocol

The energy consumption of an LPL protocol depends on the values of the parameters $I_{s},T_{e}$ and $T_{w}$, as discussed above. How long a node should sleep, wake up and remain awake in a cycle depends on the total traffic it has to process. When a new application is registered with a request for a new sensing interval or when an existing application updates its sensing request with a new sensing interval, the aggregator in the sensor-cloud determines whether or not a sensing update request is required to send to a set of physical sensors. If a new consolidated interval is found, the aggregator sends a sensing update request to the set of physical sensors, which requires the sensors to change their sensing interval accordingly. This means that the number of sensing packets produced by those source nodes will be changed, which obviously affects the scheduling of the nodes. For example, when a node updates its sensing interval with a lower value, it produces and forwards sensing packets more frequently. When the number of sensing packets generated by a source node is changed, the incoming traffic at intermediate nodes on the way to the sensor-cloud through the sink is also changed. This requires all source nodes and corresponding intermediate nodes to adapt their LPL parameters for energy optimization. Each parameter will be affected accordingly as follows when the sensing interval of a node is changed upon a sensing update request.

*The sleep interval* ($I_{s}$) : The sleep interval value indicates how frequently a node wakes up for receiving checks in a period of time. With a short sleep interval, a receiver node has to perform receive checks for incoming packets more frequently, which leads to a high energy consumption at the receiver side. However, a short sleep interval at the receiver side helps shorten the preamble transmission duration of its sender nodes, thus lowering the senders’ energy consumption. Based on this characteristic of $I_{s}$, when the updated sensing interval of source nodes is shorter than the previous one, decreasing $I_{s}$ at intermediate nodes may be a benefit to reduce the total energy consumption of senders and their receivers. With a long sleep interval, energy consumption at the receiver side is reduced, but the energy cost at the sender nodes is increased. Based on this characteristic of $I_{s}$, when the updated sensing interval of source nodes is longer and the number of packets sent by a sender is smaller, increasing $I_{s}$ may help to reduce total energy consumption.

$T_{w}$ *and* $T_{e}$: The energy consumption characteristic of $T_{w}$ and $T_{e}$ at the receiver side and the sender side is contradictory compared to $I_{s}$. When the updated sensing interval of source nodes is shorter than the previous one, increasing $T_{w}$ and $T_{e}$ at intermediate nodes may provide a benefit to energy consumption optimization as incoming traffic will increase. On the other hand, when the number of packets sent by senders is reduced, decreasing $T_{w}$ and $T_{e}$ may save energy.

### 4.6. Theoretical Framework for the Sensing Update Request-Based Adaptive LPL Protocol

We first establish a theoretical framework for the adaptive protocol. The theoretical framework captures the energy consumption characteristics of a receiver node and its sender nodes in a time window $T_{u}$. We mainly focus on the time cost of the radio wakeup of nodes, when most of the energy is consumed.

#### 4.6.1. Energy Consumption at the Receiver Side

**For receive checks:** The number of receive checks of a receiver within $T_{u}$ is $N_{rc}=T_{u}/T_{cycle}$. Denote $T_{rc}$ as the duration for a receive check. We calculate the total radio-on time cost of a receiver for receiver checks as follows.

(13) $$E_{rc}=T_{rc}T_{u}/T_{cycle}$$

**For packet receiving** (k>0): We now compute the expected receiving time cost in a cycle for a receiver. Because a receiver may lengthen its awake period when it receives a packet, the receiving time cost of a node depends on the following parameters: (1) $T_{w}$; (2) $T_{e}$; (3) the number of received packets and the inter-packet interval. Note that not all received messages trigger an extended awake period of a receiver. We define the following types of packets.

*Extending packets:* are received packets that trigger an extended awake period for the receiver. Extending packets leads to an increase in the awake period of the receiver.

*Data packets with preamble transmission:* data packets are transmitted when the receiver is still sleeping; thus, preamble transmission is required until the receiver wakes up.

*Data packets without preamble transmission:* when both the sender and the receiver are awake, packets are transmitted without a requirement of preamble transmission.

The number of received packets of a node and the inter-packet interval are random variables. Therefore, we compute the radio-on time period of a receiver based on $T_{w},T_{e}$, the probability of k extending packets and the expected inter-packet interval. The probability of k extending packets depends on the correlative value between $T_{w}$ and $T_{e}$.

***Case 1:***  $T_{w}\geq T_{e}$

**Theorem 2.**  *An extending packet should be received after* $t+I_{s}+\left(T_{w}-T_{e}\right)$.

**Proof.**  When a receiver wakes up without receiving any packet, its total wakeup period is $E_{a}=T_{w}$ from the point of time $t+I_{s}$ to $t+I_{s}+T_{w}$.

If a packet p is received at time $t_{1}$ before $t+I_{s}+\left(T_{w}-T_{e}\right)$ ($t\leq t_{1}\leq t+I_{s}+\left(T_{w}-T_{e}\right)$), the receiver will extend its awake period until at least $t_{1}+T_{e}$. However, $t_{1}+T_{e}<t+I_{s}+\left(T_{w}-T_{e}\right)+T_{e}=t+I_{s}+T_{w}$. The result indicates that receiving the packet p does not lead to an increase in the awake period of the receiver. According to the definition of an extending packet, p is not an extending packet.

If a packet p’ is received at time $t_{2}$ after $t+I_{s}+\left(T_{w}-T_{e}\right)$, the receiver will lengthen its awake period until at least $t_{2}+T_{e}>t+I_{s}+\left(T_{w}-T_{e}\right)+T_{e}=t+I_{s}+T_{w}$. The extended period is equal to $t_{2}+T_{e}-\left(t+I_{s}+T_{w}\right)>0$. According to the definition of an extending packet, p’ is an extending packet.

We use $N_{t}^{t^{\prime }}$ and $t_{i}$ to stand for the number of received packets in a time period from *t* to $t^{\prime }$ and the arrival time of packet $i_{th}$, respectively. The awake period of a receiver is lengthened if it receives at least one packet during the period from $t+I_{s}+\left(T_{w}-T_{e}\right)$ to $t+I_{s}+T_{w}$ ($N_{t+I_{s}+\left(T_{w}-T_{e}\right)}^{t+I_{s}+T_{w}}>0$).

**Theorem 3.**  *The inter-packet interval between two consecutive extending packets* $P_{\left(i+1\right)}$ *and* $P_{i}$ *should not be greater than* $T_{e}$.

**Proof.**  After receiving an extending packet $P_{i}$ at time $t_{i}$, the receiver will turn off its radio and go to sleep if it does not receive any packet during the period from $t_{i}$ to $t_{i}+T_{e}$.

If a packet $P_{k+1}$ arrives at $t_{k+1}$ with $t_{k+1}-t_{k}>T_{e}$, the receiver will not receive it because it is sleeping; thus, $P_{k+1}$ is not an extending packet.

The probability for k extending packets is calculated as follows:

(14) $$\begin{array}{cc} P_{T_{w}\geq T_{e}}\left(k\right)=P\left(N_{t+I_{s}+\left(T_{w}-T_{e}\right)}^{t+I_{s}+T_{w}}>0\right) & \overset{k-1}{\underset{i=1}{⋀}}\left(t_{i+1}-t_{i}\leq T_{e}\right)\\ & ⋀(t_{k+1}-t_{k}>T_{e}) \end{array}$$

We have $P_{T_{w}\geq T_{e}}\left(0\right)=P\left(N_{t+I_{s}+\left(T_{w}-T_{e}\right)}^{t+I_{s}+T_{w}}=0\right)$.

We now measure the expected inter-packet interval between two consecutive extending packets.

(15) $$\bar{T_{ip}\left(T_{ip}^{max}\right)}=\int_{0}^{T_{ip}^{max}}TP\left(T_{ip}=T|N_{0}^{T_{ip}^{max}}>0\right)dT$$

where $T_{ip}^{max}$ is the maximum inter-packet interval. In this case, $T_{ip}^{max}=T_{e}$. $P(T_{ip}=T)$ is the probability that the inter-packet interval is equal to T.

We now can calculate the expected total awake period $E_{a1}\left(k\right)$ of a receiver with k extending packets as follows:

(16) $$E_{a1}\left(k\right)=\left\{\begin{array}{cc} T_{w} & \;\text{if}\;k\;=\;0\\ T_{w}+k\bar{T_{ip}\left(T_{e}\right)} & \;\text{otherwise} \end{array}\right.$$

***Case 2:***  $T_{w}<T_{e}$:

**Theorem 4.**  Any packet received during the awake period of the receiver is an extending packet.

If the receiver does not receive any packet in a cycle, its total awake time period is $T_{w}$ from the point of time $t+I_{s}$ to $t+I_{s}+T_{w}$. If the receiver receives a packet at time *t*’ during its wakeup period ($t^{\prime }\geq t+I_{s}$), the receiver will set its awake period until at least $t^{\prime }+T_{e}$. Because $t^{\prime }+T_{e}\geq t+I_{s}+T_{w}$, p is an extending packet.

In other words, the wakeup period of the receiver is extended if it receives at least one packet $T_{w}$ ($N_{t+I_{s}}^{t+I_{s}+T_{w}}>0$).

Theorem 3 is also applied for this case.

We then have the probability of k extending packets:

(17) $$\begin{array}{cc} P_{T_{w}<T_{e}}\left(k\right)=P\left(N_{t+I_{s}}^{t+I_{s}+T_{w}}>0\right) & \overset{k-1}{\underset{i=1}{⋀}}\left(t_{i+1}-t_{i}\leq T_{e}\right)\\ & ⋀(t_{k+1}-t_{k}>T_{e}) \end{array}$$

The expected inter-packet interval is also computed using (16). The expected total awake period $E_{a2}\left(k\right)$ of the receiver with k extending packets in a cycle is computed as follows:

(18) $$E_{a2}\left(k\right)=\left\{\begin{array}{cc} T_{w} & \;\text{if}\;k\;=\;0\\ \bar{T_{ip}\left(T_{w}\right)}+\left(k-1\right)\bar{T_{ip}\left(T_{e}\right)}+T_{e} & \;\text{otherwise} \end{array}\right.$$

where $\bar{T_{ip}\left(T_{w}\right)}$ is the expected time period from the time the receiver wakes up to the time it receives the first packet. In case $N_{t}^{t+I_{s}}>0$, there are data packets with preamble transmission, and the first extending packet may be received immediately when the receiver wakes up; thus, $T_{ip}\left(T_{w}\right)$ can be equal to zero.

From Equations (16) and (18), we compute the expected total awake period of a receiver as follows:

(19) $$E_{a}=\left\{\begin{array}{cc} \sum_{k=0}^{\infty }E_{a1}\left(k\right)P_{T_{w}\geq T_{e}}\left(k\right) & \;\text{if}\;T_{w}\geq T_{e}\\ \sum_{k=0}^{\infty }E_{a2}\left(k\right)P_{T_{w}<T_{e}}\left(k\right) & \;\text{otherwise} \end{array}\right.$$

We now have the expected duty cycle length:

(20) $$T_{cycle}=I_{s}+E_{a}$$

From Equations (13) and (19), we compute the total radio-on time period of the receiver in a time window of $T_{u}$ as follows:

(21) $$E_{receiver}=\left(E_{rc}+E_{fw}+E_{a}\right)T_{u}/T_{cycle}$$

#### 4.6.2. Energy Consumption at Senders

The radio-on time cost of senders to send packets to the receiver depends on the total number of packets consisting of packets with preamble transmission ($N_{p}$) and non-preamble transmission ($N_{non}$).

The expected number of packets with preamble transmissions depends on $I_{s}$ and the traffic rate $R_{p}$, which is changed when the sensing interval update is requested. $N_{p}$ is computed as follows.

(22) $$N_{p}=R_{p}I_{s}T_{u}/T_{cycle}$$

The expected number of packets without preamble transmission (i.e., $N_{non}$) depends on the wakeup period of the receiver and the probability of k received packets. In case of $T_{w}\geq T_{e}$, $N_{non}$ includes packets received in a period ($t+I_{s},t+I_{s}+\left(T_{w}-T_{e}\right)$) and extending packets. In case of $T_{w}<T_{e}$, one of the received extending packets may be a packet with preamble transmission if $P(N_{t}^{t+I_{s}})>0$. Therefore, $N_{non}$ is computed as follows.

(23) $$\begin{array}{c} N_{non}=\left\{\begin{array}{c} \{\sum_{x=0}^{\infty }xP\left(N_{t+I_{s}}^{t+I_{s}+\left(T_{w}-T_{e}\right)}=x\right)+\\ \;\sum_{k=0}^{\infty }P_{T_{w}\geq T_{e}}\left(k\right)k\}T_{u}/T_{cycle}\;\text{if}\;T_{w}\geq T_{e}\\ \left\{\sum_{k=0}^{\infty }P_{T_{w}<T_{e}}\left(k\right)k-P\left(N_{t}^{t+I_{s}}>0\right)1\right\}T_{u}/T_{cycle}\\ \;\text{otherwise} \end{array}\right. \end{array}$$

We assume that the cost for sending a packet without preamble transmission is *β* s. The expected transmission duration of a packet with preamble transmission is $I_{s}/2$. The total awake period for sending packets is then:

(24) $$E_{senders}=N_{p}I_{s}/2+\left(N_{p}+N_{non}\right)\beta$$

#### 4.6.3. Expected Energy Consumption

We denote *γ*, *η* and *δ* as the energy consumption rates for the receive check, for listening/receiving and for sending. The expected energy consumption to receive and send packets is computed as follows:

(25) $$f\left(I_{s},T_{w},T_{e}\right)=E=\gamma E_{rc}+\eta E_{a}+\delta E_{senders}$$

Our goal is to optimize E to achieve the minimum energy consumption of the receiver and senders. We use Equation (25) as the guideline for our adaptive protocol design.

#### 4.6.4. Illustration to Calculate E

E can be easily obtained based on a specific distribution of traffic. We assume the traffic follows a memoryless Poisson distribution with the independence of inter-packet interval $y_{i}=t_{i+1}-t_{i}$. As intervals between events y has an exponential distribution, we have $f\left(y\right)=R_{p}e^{-R_{p}y}$. Following the Poisson distribution, we also have the probability $P\left(N_{t+I_{s})}^{t+I_{s}+T_{e}}>0\right)=1-e^{-R_{p}T_{e}}$. From the above results and Equation (14), we have:

(26) $$P_{T_{w}\geq T_{e}}\left(k\right)=P\left(N_{t+I_{s}+\left(T_{w}-T_{e}\right)}^{t+I_{s}+T_{w}}>0\right)\left(\prod_{i=1}^{k-1}\int_{0}^{T_{e}}f\left(y_{i}\right)dy_{i}\right)\int_{T_{e}}^{\infty }f\left(y_{k}\right)dy_{k}={\left(1-e^{-R_{p}T_{e}}\right)}^{k}e^{-R_{p}T_{e}}$$

(27) $$P_{T_{w}<T_{e}}\left(k\right)=P\left(N_{t+I_{s}}^{t+I_{s}+T_{w}}>0\right)\left(\prod_{i=1}^{k-1}\int_{0}^{T_{e}}f\left(y_{i}\right)dy_{i}\right)\int_{T_{e}}^{\infty }f\left(y_{k}\right)dy_{k}=\left(1-e^{-R_{p}T_{w}}\right){\left(1-e^{-R_{p}T_{e}}\right)}^{k-1}e^{-R_{p}T_{e}}$$

Similarly, we can calculate other values. The traffic rate $R_{p}$ can be measured directly. Finally, we obtain $E=f(I_{s},T_{w},T_{e})$. We later show how to use Equation (25) to optimize $I_{s},T_{w}$ and $T_{e}$ to minimize E.

### 4.7. Energy Consumption Minimization Problem

From the result of Equation (25), we formulate the energy consumption minimization problem as follows:

**Objective function:**

(28) $$\begin{array}{cccc} & \text{minimize} & & f(I_{s},T_{w},T_{e}) \end{array}$$

**Subject to:**

(29) $$I_{s}\geq 0$$

(30) $$T_{w}\geq 0$$

(31) $$T_{e}\geq 0$$

We solve the minimization problem by using the extreme value theory to find the optimal values of the LPL temporal parameters (i.e., $I_{s0},T_{w0},T_{e0}$) so that the minimum energy consumption is achieved. We have the gradient vector of *f* as:

(32) $$\overset{⟶}{▽}f=\left(\frac{\partial f}{\partial I_{s}},\frac{\partial f}{\partial T_{w}},\frac{\partial f}{\partial T_{e}}\right)$$

a vector of first order partial derivatives.

Because *f* achieves the extreme value at ($I_{s0},T_{w0},T_{e0}$), we have $\overset{⟶}{▽}f\left(I_{s0},T_{w0},T_{e0}\right)=0$. As a result, we have:

(33) $$\frac{\partial f}{\partial I_{s}}\left(I_{s0},T_{w0},T_{e0}\right)=0$$

(34) $$\frac{\partial f}{\partial T_{w}}\left(I_{s0},T_{w0},T_{e0}\right)=0$$

(35) $$\frac{\partial f}{\partial T_{e}}\left(I_{s0},T_{w0},T_{e0}\right)=0$$

By solving Equations (33)–(35) under the Constraints (29)–(31), we find the optimal values of the LPL temporal parameters ($I_{s0},T_{w0},T_{e0}$). We can check whether or not the obtained results lead to the minimum of the function *f* (i.e., the minimum energy consumption) by using the second derivation test with the Hessian matrix (H) based on the extreme value theory.

(36) $$H=\left[\begin{array}{ccc} \frac{\partial^{2}f}{\partial I_{s}^{2}} & \frac{\partial^{2}f}{\partial I_{s}\partial T_{w}} & \frac{\partial^{2}f}{\partial I_{s}\partial T_{e}}\\ \frac{\partial^{2}f}{\partial T_{w}\partial I_{s}} & \frac{\partial^{2}f}{\partial T_{w}^{2}} & \frac{\partial^{2}f}{\partial T_{w}\partial T_{e}}\\ \frac{\partial^{2}f}{\partial T_{e}\partial I_{s}} & \frac{\partial^{2}f}{\partial T_{e}\partial T_{w}} & \frac{\partial^{2}f}{\partial T_{e}^{2}} \end{array}\right]$$

where the derivatives are evaluated at ($I_{s0},T_{w0},T_{e0}$).

### 4.8. Adaptive Operations

#### 4.8.1. Traffic Rate Measurement

Theoretically, a node can calculate its incoming traffic rate based on the sensing intervals of nodes belonging to its subtree. However, this approach may be inefficient and not scalable when a subtree rooted by a node may have a great number of nodes. For efficiency, to measure the traffic rate $R_{p}$, we use a counter to count the number of incoming packets $N_{p}$ in a time window T. $R_{p}$ is then calculated by $R_{p}=N_{p}/T$. In the case of the active mode when nodes receive a sensing interval update request, traffic measurement is performed every interval of $\omega_{active}$. In lazy mode, the interval is increased to $\omega_{lazy}$, which is longer than $\omega_{active}$ for saving energy. When the traffic changes significantly, a node will perform LPL parameter adaptation to minimize its energy consumption.

#### 4.8.2. LPL Parameter Adaptation

In our LPL adaptive protocol, a receiver node adapts its LPL temporal parameters to optimize the total energy consumption of itself and its senders whenever a significant change in traffic rate is detected. The traffic rate change is normally notified by the sensing interval update request. The optimization is based on the above theoretical framework, which computes the optimal values for $I_{s},T_{w}$ and $T_{e}$. When a new set of values $(I_{s},T_{w}$ and $T_{e}$) is discovered by a node, the node then adjusts its LPL temporal parameters accordingly to optimize its energy consumption. The detailed operations for parameter calculation and exchange are discussed in [46].

## 5. Performance Evaluation

To evaluate the proposed system, we conduct extensive simulations as follows. Simulations consist of 120 sensor nodes with one sink node, one sensor-cloud and 10 different applications. The sensor-cloud is built as a Java-based web service running on a Core i5 desktop PC with 8 GB RAM, which provides sensing data to different applications. Sensing data received by the sensor-cloud are stored in a database. The SSaaS has a menu of 10 different sensing interval options from 120 s to 2 s, including the following options (120 s, 60 s, 40 s, 30 s, 25 s, 20 s, 15 s, 10 s, 5 s, 2 s). Simple applications are implemented using Java, which send sensing requests to the sensor-cloud. Each application in an ascending order (i.e., from first to 10th) joins to use the sensing service of the sensor-cloud at a random hour. When an application is registered, it sends a sensing request to the web service with a selected sensing interval among the options recommended by the sensor-cloud. For fairness, each application will select a sensing interval randomly.

We use three types of sensors, including temperature sensors, humidity sensors and pressure sensors. Each type of sensor consists of 40 nodes, which are deployed randomly and use the same sampling frequency. Each sensor belonging to a type is assigned with a multicast address. We use multicast to disseminate sensing interval update requests to specific physical sensors. Each application is assumed to request a type of sensing data among the three above. Virtual sensors are implemented as objects in the web service. Application requests can be encoded in the form of XML templates, which are decoded by the SensorML interpreter [48,49]. Based on application requests, the SSaaS allocates corresponding virtual sensors to the applications. If the request aggregator determines the need for a sensing interval update for corresponding physical sensors, an HTTP-based request will be sent to the sink node where the HTTP-CoAP converter [50] presented in our previous work will convert the HTTP request to a CoAP request. The request is then sent to corresponding physical sensor nodes.

Collection tree protocol (CTP) is used as a data collection protocol [51] for physical sensor nodes. According to CTP, sensor nodes form a tree-based topology toward the sink node. Sensing data are gathered at the sink node; then, the sink forwards data packets to the sensor-cloud to serve applications. All schemes use LPL [49] in the lower layer for energy efficiency. We implement our adaptive LPL on the top of the existing TinyOS LPL MAC protocol [49]. The implementation consists of three main components: traffic rate estimator, parameter optimizer and duty cycling adapter. The traffic rate estimator operates based on the triggering event of sensing interval update requests. If the estimator detects a significant change (i.e., a change is over 5%) in the traffic, it triggers a call to the parameter optimizer. The parameter optimizer performs a calculation for the optimal values of LPL temporal parameters. To avoid complexity, we pre-compute the optimal values for those parameters under different rates corresponding to the range of sensing interval options offered by the sensor-cloud. Each node stores those values locally. The parameter optimizer uses those values to search for the optimal values of the LPL parameters in each specific case. If the optimizer finds any change in the optimal setting, it calls the adapter to adjust the duty cycle parameters accordingly.

### 5.1. System Configuration

To ensure realistic simulation evaluation, we use the radio noise model based on closest-fit-pattern matching (CPM) and an experimental noise trace (i.e., meyer-heavy.txt) from the Meyer Library at Stanford University [52]. We keep default CCA checks of the TinyOS LPL up to 400 times. Table 2 presents the detailed parameters used in our simulations. Other parameters are set to the default values of the TOSSIM radio model for CC2420.

### 5.2. Performance Metrics

We evaluate the system in terms of: (1) energy efficiency; (2) bandwidth consumption; (3) delay; (4) reliability; and (5) scalability

*Energy efficiency*: we use the average radio duty cycle as an indicator of energy efficiency, because most of the energy in a sensor node is consumed by its radio module.

*Bandwidth consumption*: we measure the number of messages forwarded between the sink and the sensor-cloud to indicate the bandwidth consumption of the system.

*Delay*: Because the sink-to-sensor-cloud packet delivery delay is normally negligible compared to the packet delay in constrained sensor networks, we report only the sensor-to-sink data packet delivery delay [53].

*Reliability*: we measure the ratio between the number of successful delivered packets to the sink node and the total number of forwarded packets to indicate the reliability of the system.

*Scalability*: we test the system with various numbers of applications to show the scalability of the system.

### 5.3. Results

We compare the proposed system to (1) dedicated application requests with in-network data packet aggregation (D-aggregation), where in-network aggregation [11] is applied for sensing traffic to improve energy efficiency; and (2) dedicated application requests with multi-task optimizations (D-opt) [12], where each sensing request sent to physical sensors is treated as a task and multi-task optimization techniques are applied to improve efficiency. We report the average results of 100 different runs.

#### 5.3.1. Dynamic Number of Applications and Traffic Loads

**Figure 4:**
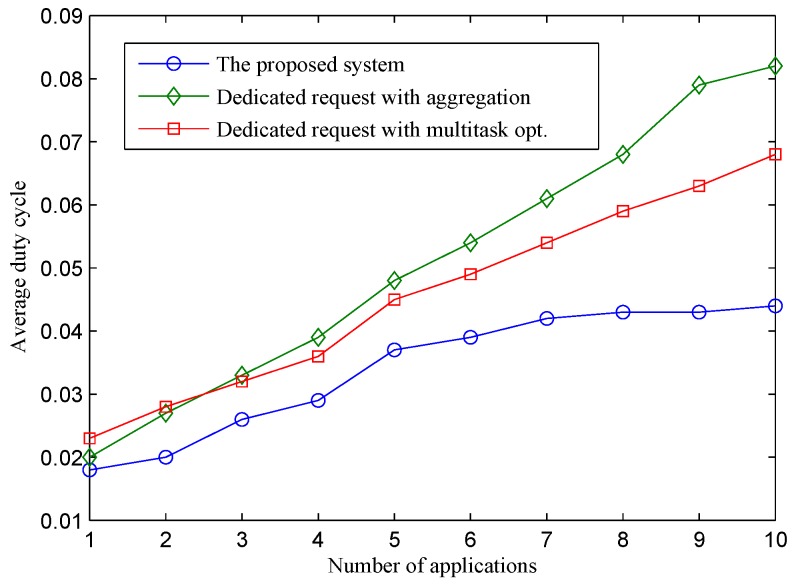
Average duty cycle vs. the number of applications.

Figure 4 presents the average duty cycle of the three approaches under a different number of applications. The duty cycle of physical nodes using the two dedicated sensing request approaches increases quickly when the number of applications increases. When a new application that requests a new sensing interval joins the sensor-cloud, sensors are requested to perform an additional sensing task to serve the application, which incurs more workload for constrained sensor devices. A single sensor node may have to run multiple sensing tasks to serve different applications. This means that the node has to wake up more frequently to perform sensing and transmit sensing packets. As a result, its energy consumption is highly proportional to the number of applications. In the D-opt approach, multi-task optimization [12] is performed to reduce the number of sensing samplings. This technique helps D-opt achieve a better energy efficiency compared to D-aggregation. By performing request aggregation on the sensor-cloud, the proposed approach achieves the highest energy efficiency. In particular, the duty cycle of sensor nodes is maintained under 5%, even if they have to serve up to 10 different applications. The result indicates that the duty cycle of sensor nodes is not impacted much by the number of applications. This is due to the fact that the proposed system enables each sensor to run only a single sensing task with a consolidated sensing interval, which satisfies all of the applications, thus reducing the workloads for physical sensor nodes considerably. The proposed system achieves a significant improvement in terms of energy efficiency compared to the two dedicated approaches. The improvement increases proportionally with the number of applications. Within one application, the duty cycle of the proposed system is only slightly lower than that of the two approaches, because the scheduling of sensor nodes in the proposed system is adapted to minimize energy consumption. When the number of applications increases to 10, the improvement of the proposed system increases to 54% compared to D-opt, and 86% compared to D-aggregation.

**Figure 5:**
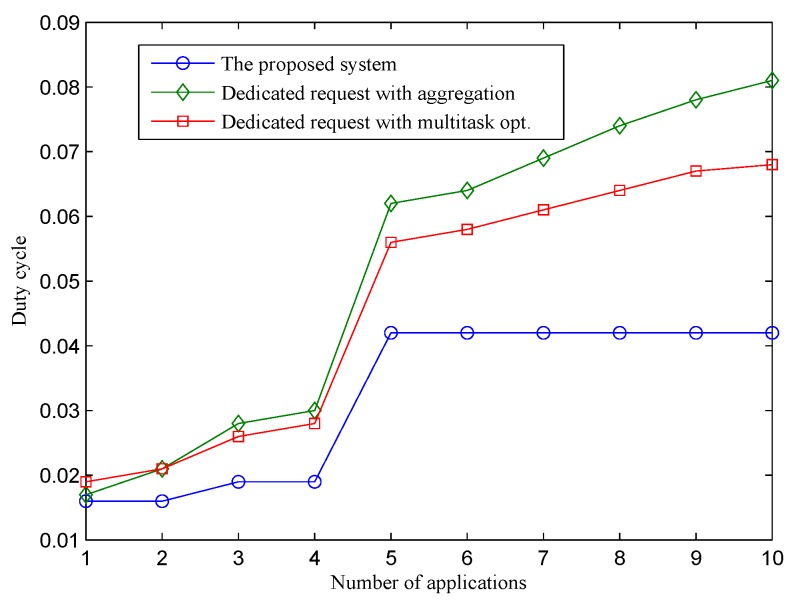
Average duty cycle vs. the number of applications that request sensing intervals as follows: 60 s, 120 s, 30 s, 40 s, 2 s, 5 s, 10 s, 15 s, 20 s and 25 s, respectively.

In Figure 5, we show the duty cycle performance of the proposed system in a specific test case where sensing intervals requested by applications from one to 10 are 60 s, 120 s, 30 s, 40 s, 2 s, 5 s, 10 s, 15 s, 20 s and 25 s, respectively. The purpose of showing this figure is to provide an understanding about the performance behaviors of the proposed system in relation to different sensing interval requests from applications. The figure shows clearly that the performance of our system does not depend on the number of applications, but the traffic load of sensor nodes. Within the first application that has requested a sensing interval of 60 s, the duty cycle of sensor nodes is about 1.6% on average. When the second application with a requested sensing interval of 120 s is deployed, the duty cycle of the sensor nodes does not change. Sensing interval requests sent by the second application are hidden from sensor nodes because the request aggregator determines that sensing requests by the second application can be satisfied by the current consolidated sensing interval. As a result, there is no change in physical sensor nodes. When the third application is deployed with a requested sensing interval of 30 s, the current consolidated sensing interval is longer and does not satisfy the requirements of the third application. The request aggregator finds a new consolidated sensing interval and sends a sensing update request to corresponding sensor nodes. With a shorter sensing interval, the traffic load of sensors increases, and the system adjusts their LPL parameters to adapt to the new traffic condition (i.e., shorten $I_{s}$). As a result, the average duty cycle of sensor nodes increases from 1.6% to 1.9%. Similar to the case of the second application, sensing requests by the fourth application are hidden from physical nodes. The fifth application has a much shorter requested sensing interval, which leads to a considerable increase in the sensing traffic of sensor nodes. As a result, the average duty cycle result jumps to 4.2%. After that, the average duty of sensor nodes remains stable at this value. The consolidated sensing interval of sensor nodes does not change because later applications (6th, 7th, 8th, 9th and 10th) have requested sensing intervals longer than the current consolidated sensing interval. The current consolidated sensing interval can satisfy all of the later applications. While the duty cycle of the proposed system does not change when the 6th, 7th, 8th, 9th and 10th applications are deployed, the duty cycles of the two dedicated approaches keep increasing considerably. In terms of energy consumption, the scalability and energy efficiency of the two dedicated approaches are quite low. The reason is that if there are many applications requesting such a sensing service, the network lifetime of the physical sensor nodes will be short. This incurs a high cost and difficulty in sensor deployment for sensor owners. Through this experiment, we conclude that the energy consumption of the proposed system does not depend on the number of applications, but the length of the consolidated sensing interval instead. This characteristic of the proposed system enables a single sensor network to serve multiple applications with a controllable expected lifetime (i.e., by defining allowable sensing interval options).

**Figure 6:**
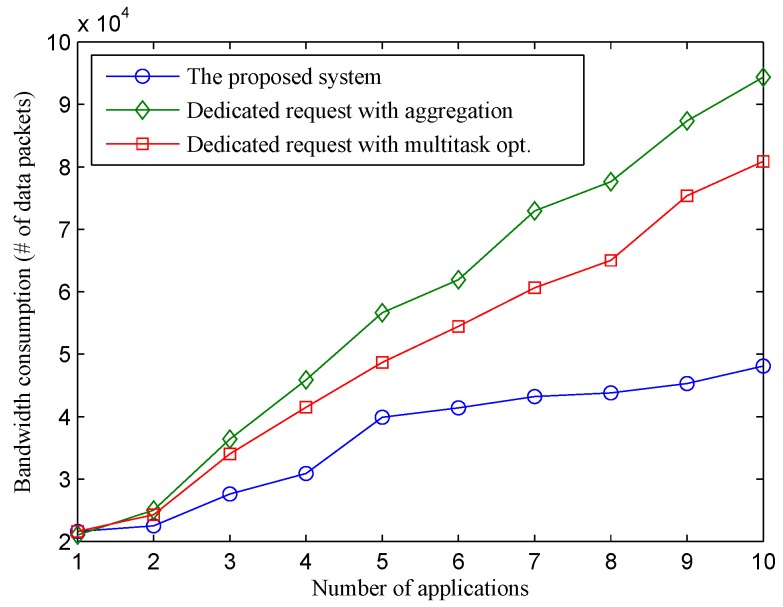
Bandwidth consumption vs. the number of applications.

Physical sensor utilization and bandwidth consumption are two main factors in calculating the price of sensor-cloud services [4]. Figure 6 shows the bandwidth consumption of different approaches in correlation with the number of applications. Within one application, the bandwidth utilization of the three approaches is similar. When the number of applications increases, the bandwidth consumption of the two dedicated approaches increases rapidly. Although sensing traffic optimization using aggregation or task optimization is applied, the number of packets forwarded between the sink node and the sensor-cloud in the two dedicated approaches is still much higher compared to that of our proposed system. The gap between the bandwidth consumption of the proposed system and the two dedicated schemes increases proportionally to the number of applications. The reason is that while the traffic load of sensor nodes in the two dedicated schemes increases significantly when a new application with a new sensing interval is deployed, the traffic load of sensor nodes in the proposed system is not impacted by the number of applications, but by the length of the consolidated sensing interval, as shown in Figure 5. Sensing requests from new applications with a longer sensing interval compared to the consolidated sensing interval are hidden from sensor nodes, thus saving a considerable amount of bandwidth. When all ten applications are deployed, the bandwidth consumption of the two dedicated schemes is almost double the bandwidth consumption of the proposed scheme. The results also indicate that the proposed request aggregation technique on the sensor-cloud is even more efficient compared to in-network aggregation and multi-task optimization and can be a promising technique to complement in-network aggregation to save network resources.

**Figure 7:**
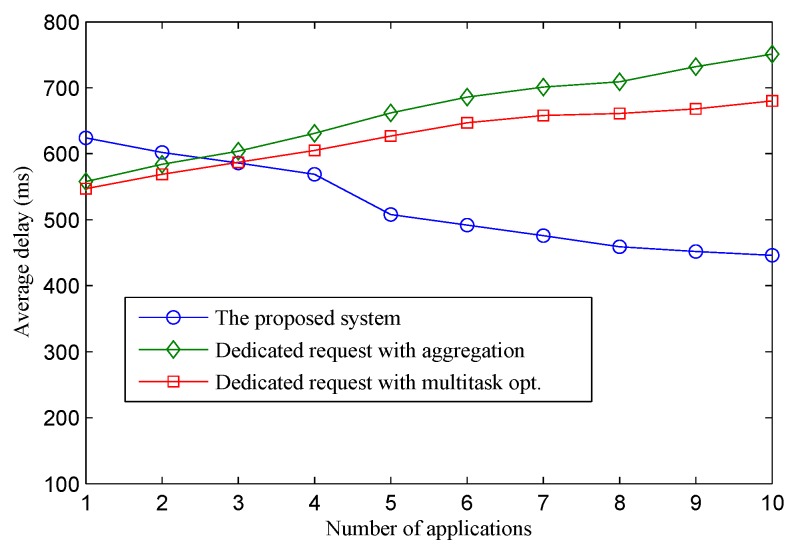
Average packet delivery delay vs. the number of applications.

Packet delivery delay in wireless sensor networks is highly proportional to the traffic load due to the narrow band usage. Figure 7 illustrates the correlation between the average packet delivery delay of the sensor-to-sink traffic with the number of applications. Within a few applications with low sensing traffic deployed, the packet delay of the proposed system is slightly higher than the other two schemes. This is because the adaptive mechanism in the proposed system automatically adapts the LPL temporal parameters in a low traffic condition (i.e., lengthen $I_{s}$) to save energy. However, when network traffic increases due to the deployment of more new applications with shorter requested sensing intervals, packet delivery delay in the proposed system significantly decreases. The reason is that the adaptive protocol adjusts the LPL temporal parameters (i.e., shorten $I_{s}$) to adapt to the new and high network traffic condition. When the network traffic increases (i.e., notified by new sensing update request), the proposed adaptive protocol automatically shortens the sleep interval of sensor nodes accordingly to quickly forward packets to save the energy of senders and reduce the overall packet delay. Even though multiple applications are deployed, the traffic load of sensor nodes is not exceeded, as requested sensing intervals from different applications are aggregated into a single consolidated sensing interval; thus, high traffic load is not a serious problem impacting on the packet delay in our proposed system. On the contrary, packet delivery delay in the two dedicated approaches is highly impacted by the number of deployed applications. Each deployed application puts more traffic load toward the sensor nodes. As a result, a node has to forward more and more packets within a cycle while scheduling parameters are fixed and channel bandwidth is limited. This is the main reason that leads to a significant increase in packet delay when the number of applications increases.

**Figure 8:**
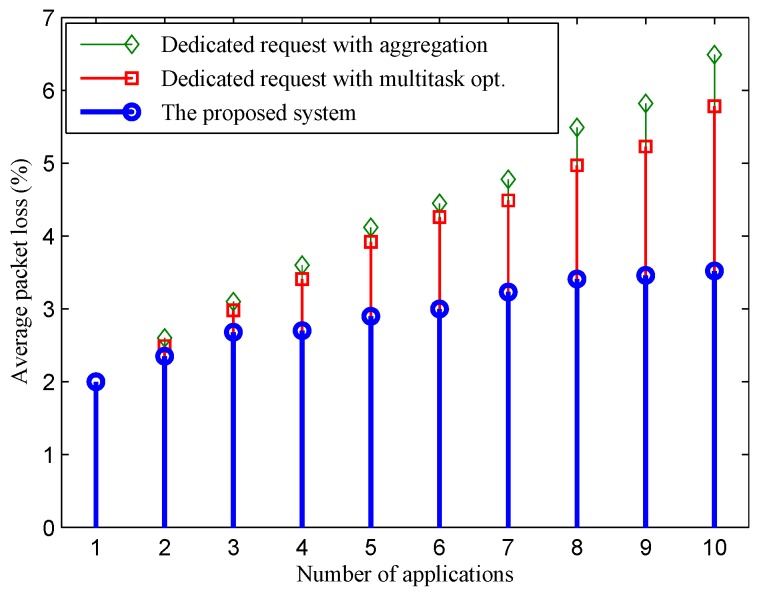
Average packet loss ratio vs. the number of applications.

Rapid traffic load increasing when the number of applications increases also causes a considerable increment in the packet loss of the two dedicated schemes, as shown in Figure 8. Within one application, the packet loss ratio witnessed for all of the schemes is similar. However, when the number of deployed applications increases, the gap between packet loss ratios of the proposed system and the two other schemes is widened. By comparing Figure 6 and Figure 8, we see that the packet loss ratio of a scheme is highly correlated with its traffic load. Note that the bandwidth consumption also reflects the total traffic load of sensor nodes. With the excessive increase in the traffic load of each sensor when more applications are deployed under fixed scheduling parameters and limited channel bandwidth, packet loss ratio graphs of the two dedicated schemes grow noticeably. In the case of the proposed system, although when a new shorter consolidated interval is found and the traffic load also increases accordingly, the packet loss ratio remains at a low level. This is due to the following reasons: (1) the traffic load increasing of a node in the proposed system is negligible compared to the two dedicated approaches; and (2) when traffic load increases, the adaptive protocol adapts the scheduling parameters to enable a node to provide a higher capability in forwarding packets.

#### 5.3.2. Scalability Test

**Figure 9:**
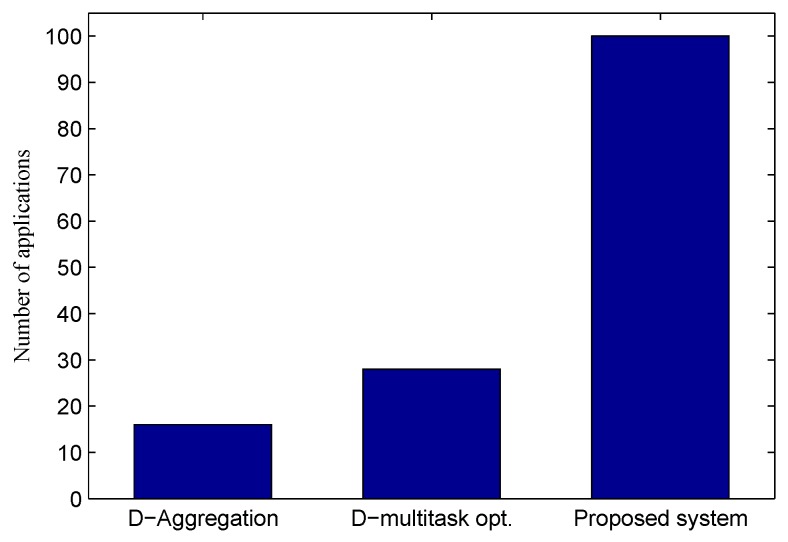
Scalability test.

We are now interested in evaluating the scalability of the systems. To compare the scalability of different schemes, we define a reliability requirement by assuming a lower bound value for the successful packet delivery ratio of sensing data packets within 90%. For each scheme, we do experiments by increasing the number of applications until the reliability of the scheme falls down lower than 90%. Each application randomly selects a sensing interval in the range of [2 s, 120 s]. The scalability of the scheme is defined as the number of applications that the scheme can support while the reliability requirement is achieved. Results are reported in Figure 9. The D-aggregation scheme can support only 16 applications with different requested sensing intervals. Note that in the dedicated approaches, the sensor-cloud enables the reusability of sensing traffic for only applications with the same requested sensing interval. D-opt has a better scalability with 28 applications. The two schemes show a limited scalability because their traffic load is proportional to the number of applications. The proposed system achieves the highest scalability among the three schemes. In particular, the system still achieves the reliability requirement when 100 applications with different sensing intervals are deployed. In fact, the scalability of the system does not depend on the number of applications. With the lower bound of a sensing interval of 2 s, we believe the sensor network can support an unlimited number of applications as long as the sensor-cloud scales well. We find that the scalability of the proposed system depends more on the minimum consolidated sensing interval instead. To support this statement, we extend the sensing interval range by decreasing the lower bound of the sensing interval to 0.5 s. We find that the reliability of the system is reduced significantly when a number of applications request the sensing interval at 0.5 s.

#### 5.3.3. Economics of the Model

According to the pricing model of the sensor-cloud [4,24], applications can request sensing services on-demand, and they are priced as usage sensitive or the pay-per-use model based on the costs of sensor usage and cloud usage cost. The economics of our system is justified as follows. Firstly, the proposed system helps reduce the cost of sensor usage by reducing the number of sensing requests sent to sensor nodes and the energy consumption of sensor nodes per application. Many application sensing requests can be hidden from sensor nodes by the request aggregator. As a result, the proposed system improves the network lifetime and, thus, reduces the cost of sensor network ownership. Secondly, the proposed system helps decrease the bandwidth consumption significantly to serve the same number of applications compared to the current approaches. Third, by achieving a high scalability, the proposed system enables the sensor-cloud to sell sensing services of a given physical sensor network to a greater number of applications while still satisfying requirements of all applications. This definitely enables sensor-cloud providers and sensor owners to make more profits and helps to cut the price of a sensing service for an application. As a result, our proposed system enables a win-win model for sensor-cloud providers, sensor owners and application owners.

## 6. Discussion and Conclusions

This paper presents an efficient interactive model for the sensor-cloud to provide sensing services for multiple applications on-demand. In the interactive model, both the cloud and physical sensor nodes are necessarily involved. The model highlights the role of the cloud in optimizing workloads for constrained physical nodes while guaranteeing that the requirements of all applications are satisfied. For that purpose, an aggregator and a request aggregation scheme on the sensor-cloud are proposed, which minimize the number of requests sent to physical sensor nodes, minimizing the number of sensings and the number of sensing packet transmissions required for physical sensor nodes. On the physical sensor side, sensors perform sensing tasks based on the guidance of the sensor-cloud. Based on the interactions with the sensor-cloud, a sensing request-based adaptive LPL protocol is proposed to minimize the energy consumption of constrained sensors. Through extensive analysis experiments, we show that the proposed system achieves a significant improvement in terms of network performance and scalability compared to current approaches and enables a win-win model for the sensor-cloud. In this version, the model is presented using sensing frequency (i.e., sensing interval) requests as an example. However, the model can be generalized for any application requirement. In future work, we are going to investigate the model for up-stream sensing traffic for packet delivery latency and reliability guarantees based on on-demand application requests.

## Figures and Tables

**Table 1:** List of symbols.

Parameter	Meaning
$I_{se}^{\alpha }$	Dedicated sensing interval of application *α*
$I_{se}^{c}$	Consolidated sensing interval
*τ*	Sensor type
RI	Region of interest
Traffic rate ($R_{p}$)	The number of incoming data packets in a unit of time (i.e., 1 s)
Sleep interval ($I_{s}$)	The sleep period in a cycle
Active period ( $E_{a}$)	The total wakeup period in a cycle, which depends on the following two parameter
Periodic wakeup period ( $T_{w}$)	The period a node remains awake after waking up in every cycle if the node does not send or receive any packet
Extended wakeup period ($T_{e}$)	The extra period a node extends its wakeup time after receiving a packet
Cycle length ($T_{cycle}$)	The period between two consecutive sleep times; $T_{cycle}=I_{s}+E_{a}$
Number of received packets (k)	The number of packets a node receives in a cycle during its active period.

**Table 2:** Parameters.

Parameter	Value	Parameter	Value
Number of cloud	1	Sensing interval options	[2 s, 120 s]
Number of sensors	120	Number of sink node	1
Number of applications	10	Number of sensor types	3
Data packet length	32 bytes	Preamble packet length	9 bytes
Time window T	10 s	CCAchecks	Up to 400 times
$I_{s}^{TinyOS-LPL}$	0.5 s	Hardware	CC2420
$T_{w}^{TinyOS-LPL}$	10 ms	$\omega_{active}$	2 s
$T_{u}$	1 s	$\omega_{lazy}$	120 s
$I_{s}^{TinyOS-LPL}$	0.5 s	$T_{e}^{TinyOS-LPL}$	100 ms
*γ*	18.8 mA	*η*	18.8 mA
*δ*	17.4 mA	Transmission range	20 m
